# Evaluating Glucagon-Like Peptide-1 Receptor Agonist Safety Before Upper Endoscopy: A Systematic Review and Meta-Analysis

**DOI:** 10.14740/gr2108

**Published:** 2026-04-27

**Authors:** Zohaib Ahmed, Amna Iqbal, Sami Ghazaleh, Abdallah Kobeissy, Ali Nawras, Yaseen Alastal, Hani Saad, Sarmed Mansur, Idrees Mohiuddin, Umair Iqbal, Dushyant Singh Dahiya, Syeda Faiza Arif, Connor Campbell, Wade Lee-Smith, Mohammad W. Asif, Faisal Kamal, Aamir Saeed, Omar Horani

**Affiliations:** a Division of Gastroenterology and Hepatology, University of Toledo College of Medicine and Life Sciences, Toledo, OH, USA; b Division of Hospital Medicine, University of Toledo College of Medicine and Life Sciences, Toledo, OH, USA; c Division of Gastroenterology and Hepatology, WellSpan Health, York, PA, USA; d Division of Gastroenterology and Hepatology, University of Kansas, Lawrence, KS, USA; e Dow University of Health Sciences, Karachi, Pakistan; f University of Toledo Medical Science Health Campus, Toledo, OH, USA; g University Libraries, University of Toledo, Toledo, OH, USA; h Department of Neuroscience, Ohio State University, Columbus, OH, USA; i Jefferson Health-Thomas Jefferson University Hospitals, Philadelphia, PA, USA; j Vanderbilt University Medical Center, Nashville, TN, USA

**Keywords:** Glucagon-like peptide-1 receptor agonists, Retained gastric contents, Upper endoscopy, EGD

## Abstract

**Background:**

The use of glucagon-like peptide-1 receptor agonists (GLP-1 RAs) has remarkably increased in the past few years as they have demonstrated significant reduction in A1c and major cardiovascular outcomes and have led to weight loss with low risk of hypoglycemia. GLP-1 RA use has raised concerns about potential associations with increased gastric retention and adverse outcomes in upper endoscopy procedures.

**Methods:**

We conducted a comprehensive search across multiple databases to identify comparative studies evaluating the impact of GLP-1 RA exposure versus non-exposure on upper endoscopy outcomes. Studies comparing GLP-1 RAs to other medications were excluded. The primary outcome was the presence of retained gastric contents, with secondary outcomes including aspiration risk and the need for repeated esophagogastroduodenoscopy (EGD).

**Results:**

Our systematic review included 28 studies, comprising data from 212,082 individuals collected between 2015 and 2023. The meta-analysis revealed that GLP-1 RA use was associated with approximately four times higher odds of retained gastric contents compared to non-exposure (odds ratio (OR): 3.95, confidence interval (CI): 2.691–5.815, P < 0.0001, I^2^: 82.71%). There were no increased odds of aspiration (OR: 1.09, CI: 0.459–2.600, P = 0.145, I^2^: 91.44%) or repeated upper endoscopies (OR: 1.82, CI: 0.813–4.085, P = 0.145, I^2^: 85.32%). However, GLP-1 RA use was significantly associated with increased odds of procedure cancellation (OR: 3.96, CI: 2.870–5.563, P < 0.0001, I^2^: 4.19%).

**Conclusion:**

While GLP-1 RAs do not increase the risk of aspiration or necessitate repeated procedures, they are linked to a higher incidence of retained gastric contents and procedure cancellations. Further randomized controlled trials are necessary to better understand the effects of GLP-1 RAs on upper endoscopy outcomes.

## Introduction

Glucagon-like peptide-1 receptor agonists (GLP-1 RAs) are widely used for management of type 2 diabetes mellitus (T2DM) and obesity due to their effectiveness in regulating serum blood glucose levels and promoting weight loss [1]. Common side effects of GLP-1 RAs include nausea, vomiting, diarrhea, and constipation [2]. Given their cardiometabolic benefit [3, 4] and weight loss effects [5], their global usage has increased from 1.2% in 2014 to 15% in 2022 in patients with type 2 diabetes mellitus and cardiovascular risk factors [6, 7].

GLP-1 RA use prior to upper endoscopy has raised concerns regarding the safety of these medications [8–10]. According to available data, aspiration and unintentional intubations pose a potential hazard due to risk of aspiration pneumonia and high rates of in-hospital mortality [11, 12]. Moreover, retained gastric contents (RGCs) and the need for repeat endoscopy are significant concerns, as they can impair procedure visibility and lead to unnecessary utilization of healthcare resources [13].

With the rise of GLP-1 RA use, the American Society of Anesthesiologists released a statement [14] advising consideration of holding GLP-1 RA prior to endoscopy, which prompted a multi-gastroenterology society statement [15, 16] concluding that there were no actionable data to suggest stopping GLP-1 RA prior to endoscopy. This illustrated competing concerns regarding GLP-1 RA use prior to endoscopy. Several more recent studies have emerged [17–19] identifying a correlation between gastric residue on esophagogastroduodenoscopy (EGD) and GLP-1 RA use.

Despite the rapid expansion of GLP-1 RA use and growing concerns regarding delayed gastric emptying, there remains a lack of high-quality, consolidated evidence evaluating their impact on clinically relevant endoscopic outcomes, including RGCs, aspiration risk, and procedure cancellation. This knowledge gap contributes to ongoing uncertainty in peri-procedural management and inconsistent recommendations regarding whether these agents should be withheld prior to endoscopy. This study aims to systematically review the literature and perform a meta-analysis to evaluate the association between GLP-1 RA use and endoscopic outcomes, including RGCs, aspiration events, procedure cancellation, and repeat endoscopy.

## Materials and Methods

### Search strategy

A comprehensive search strategy to identify studies reporting the effects of GLP-1 agonists on stomach emptying or mucosal visibility in EGD procedures was constructed in Embase (Embase.com, Elsevier) by an experienced health sciences librarian (WL-S) using truncated keywords, phrases, and subject headings. This strategy was translated to MEDLINE (PubMed platform, National Center for Biotechnology Information, National Library of Medicine), Cochrane Central Register of Controlled Trials (CochraneLibrary.com, Wiley), and the Web of Science Core Collection (Web of Science platform, Clarivate) with all searches performed on June 25, 2024 (Supplementary Material 1, gr.elmerpub.com). No publication date or language limits were used. All results were exported to EndNote 21 citation management software (Clarivate, Philadelphia, PA, USA) and duplicates were removed by successive iterations of EndNote’s duplicate detection algorithms and manual inspection. We adhered to Preferred Reporting Items for Systematic Reviews and Meta-Analyses (PRISMA) guidelines [20] (Supplementary Material 2, gr.elmerpub.com). No language restrictions were applied.

### Inclusion and exclusion criteria

Two study authors (ZA and SD) reviewed records. Animal studies, pediatric populations, review articles, case reports, and case series were excluded. Studies that did not assess outcomes related to upper endoscopy, single arm studies without a control group, and studies comparing GLP-1 RAs to other medications were also excluded. Studies that met the following criteria included randomized controlled trials and prospective or retrospective cohort studies that compared endoscopy outcomes with or without prior exposure to GLP 1-RA, and reported outcomes of interest. Abstracts were included given limited availability of literature on this topic prior to 2022 to minimize publication bias [21]. Inclusion of each study was agreed upon by two authors (ZA and SD) and a third reviewer (AI) resolved any discrepancies in study inclusion.

### Data extraction and study outcomes

The following data were extracted from the studies: author name, publication year, country of origin, study design, gender and age of patients, and total upper endoscopy procedures (Supplementary Material 3, gr.elmerpub.com). Outcome measures were retrieved with RGCs as the primary outcome. Most studies reported an adjusted odds ratio (OR), which was recorded; otherwise, the number of patients with RGC over total events was recorded. Secondary outcomes included aspiration events (aspiration, aspiration pneumonia, and aspiration pneumonitis), as reported by individual studies. Due to inconsistent and non-uniform reporting, these outcomes were pooled as a composite “aspiration events” outcome. Other outcomes included number of repeat endoscopy procedures and procedure cancellations, which were extracted and recorded as events of interest over total number of procedures (Supplementary Material 4, gr.elmerpub.com). Of note, events of interest and total number of procedures from propensity-matched comparison groups were recorded to limit confounders. Data were collected and tabulated on Microsoft Excel (Microsoft, USA) by two independent reviewers (AI and SD). Any discrepancy in data collection was resolved through mutual discussion with a third reviewer (ZA). Definitions of RGCs were not fully standardized across included studies. In most studies, RGC was defined as the presence of visible food residue in the stomach at the time of upper endoscopy despite adherence to standard fasting protocols. However, some studies categorized RGC qualitatively (e.g., minimal residue vs. significant retained contents) or based on procedural impact (e.g., impaired visualization or procedure interruption). This variability in outcome definition was considered during data interpretation. When available, information regarding timing of the last GLP-1 RA dose prior to endoscopy was extracted; however, this was inconsistently reported across studies. When available, information regarding timing of GLP-1 RA use prior to endoscopy was extracted; however, this was inconsistently reported across studies. For studies reporting both unmatched and propensity-matched cohorts, data from the propensity-matched cohorts were preferentially extracted and included in the meta-analysis to minimize confounding. Unmatched cohorts from the same studies were not included to avoid duplication.

### Data synthesis and analytical analysis

Comprehensive Meta-Analysis (CMA) Software Version 4 (Bio STAT, Englewood, New Jersey, USA) was used to perform meta-analysis. For the primary outcome of RGC, reported adjusted ORs and CIs were entered as reported by the study; otherwise the ORs and CIs were calculated from events of interest over the total number of procedures. ORs and CIs were pooled to determine an overall effect size. Several included studies reported adjusted ORs while others only provided event data from which crude ORs were calculated. To maximize inclusion of available evidence, both adjusted and calculated crude ORs were pooled using a random-effects model. For secondary outcomes, events of interest over the total number of procedures were used to calculate ORs and CIs that were pooled to determine an overall effect size. The random effects model was used with a confidence level of 95%. Statistical significance was defined as a P-value of < 0.05. Heterogeneity was assessed using the Higgins I^2^ index, where I^2^ values > 50% implied the presence of substantial heterogeneity [22].

For outcomes with 10 or more studies, a funnel plot was generated, and visual symmetry was assessed to comment on publication bias for each outcome (Supplementary Material 5, gr.elmerpub.com).

For the primary outcome, data from greater than three peer-reviewed manuscripts were available, so we conducted sensitivity analysis for RGC excluding abstracts (Supplementary Material 6a, gr.elmerpub.com). Additionally, for outcomes with 10 or more studies including RGC and cancelled procedures, sensitivity analysis was performed excluding each study individually to assess the resultant effect size (Supplementary Material 6b, c, gr.elmerpub.com). Sensitivity analysis was performed to assess the robustness of pooled effects. No subgroup analyses were performed compared to individual GLP-1 RAs as most studies assessed a mixture of GLP-1 RA agents compared to a control.

### Bias assessment

The bias assessment for included studies was evaluated using Newcastle-Ottawa scale for observational studies [23]. Publication bias was visually assessed using funnel plots and quantitatively assessed using Egger’s regression analysis. A P-value < 0.05 was indicative of substantial publication bias. Publication bias was visually assessed using funnel plots and quantitatively assessed using Egger’s regression analysis. A P-value < 0.05 was indicative of substantial publication bias.

## Results

### Study selection

**Figure 1:**
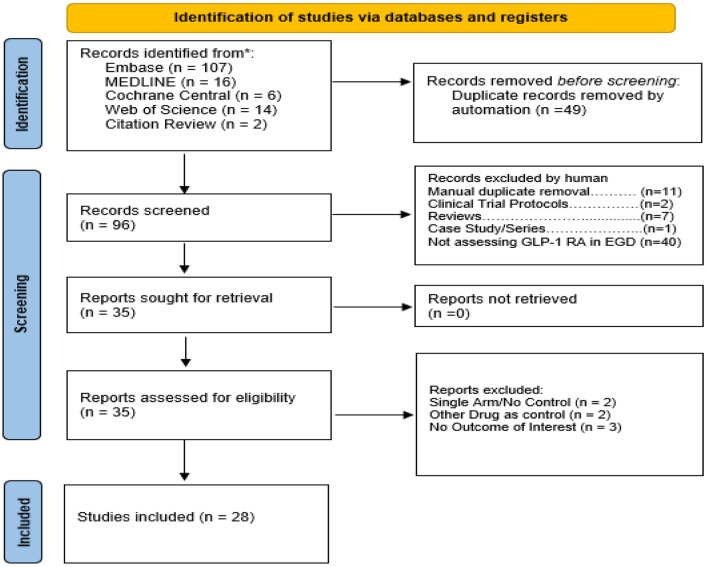
PRISMA 2020 flow diagram for systematic review if databases, registers, and other sources.

The search strategy produced 145 articles, 49 of which were removed as duplicates (PRISMA flow chart, Fig. 1). Of the remaining 96 articles, 61 were removed after title and abstract review. No additional relevant articles were identified from a review of bibliographies. After the full texts of the remaining 35 articles were reviewed, we excluded two non-comparative studies. Three studies were excluded as they did not include relevant data. Two studies were excluded as they were case reports. A total of 28 retrospective studies [8, 10, 24–48] including 21 cohort studies, six case-control studies, and one cross-sectional study were included in final analysis.

### Study characteristics

Our final analysis included 212,082 patients with an average mean reported age of 56.2 years, 60.1% of the population were female, 56.3% had T2DM, and mean BMI was 34.2. Four studies assessed semaglutide alone with the remaining studies reporting a mixed group of GLP-1 RAs. All studies included patients who had GLP-1 exposure of at least 3–4 months.

### Meta-analysis

#### Primary outcome

**Figure 2:**
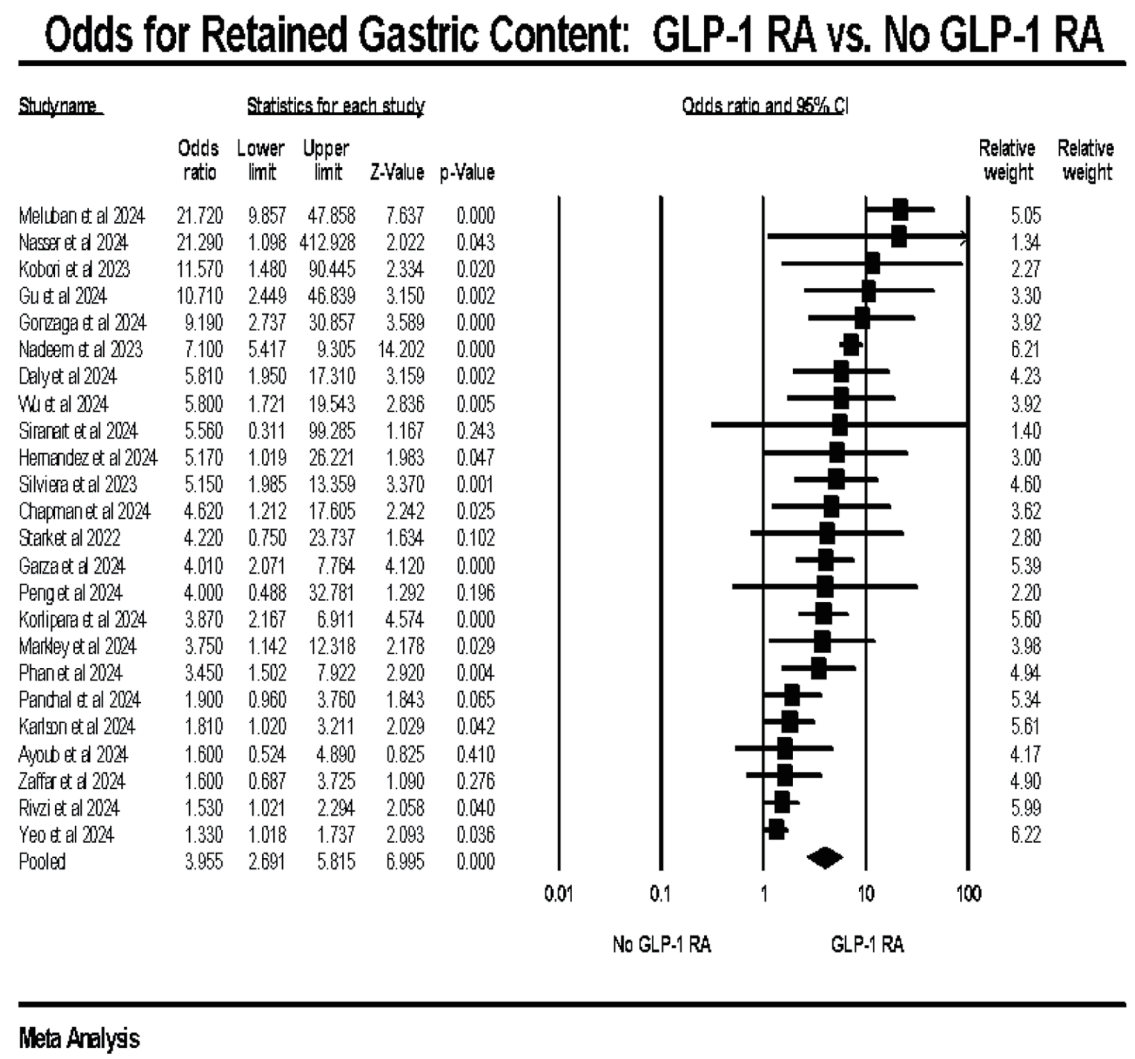
Primary outcome: odds of retained gastric content in GLP-1 RA vs. no GLP-1 RA exposure. GLP-1 RA: glucagon-like peptide-1 receptor agonist.

Our pooled analysis revealed a higher odd of RGC in the GLP-1 RA group compared to the group with no prior exposure to GLP-1 RA (OR: 3.95, CI: 2.691–5.815, P < 0.0001, I^2^: 82.71%) (Fig. 2). Across included studies, the reported incidence of retained gastric contents varied, with higher rates observed in patients receiving GLP-1 RAs compared to non-exposed patients.

#### Secondary outcomes

**Figure 3:**
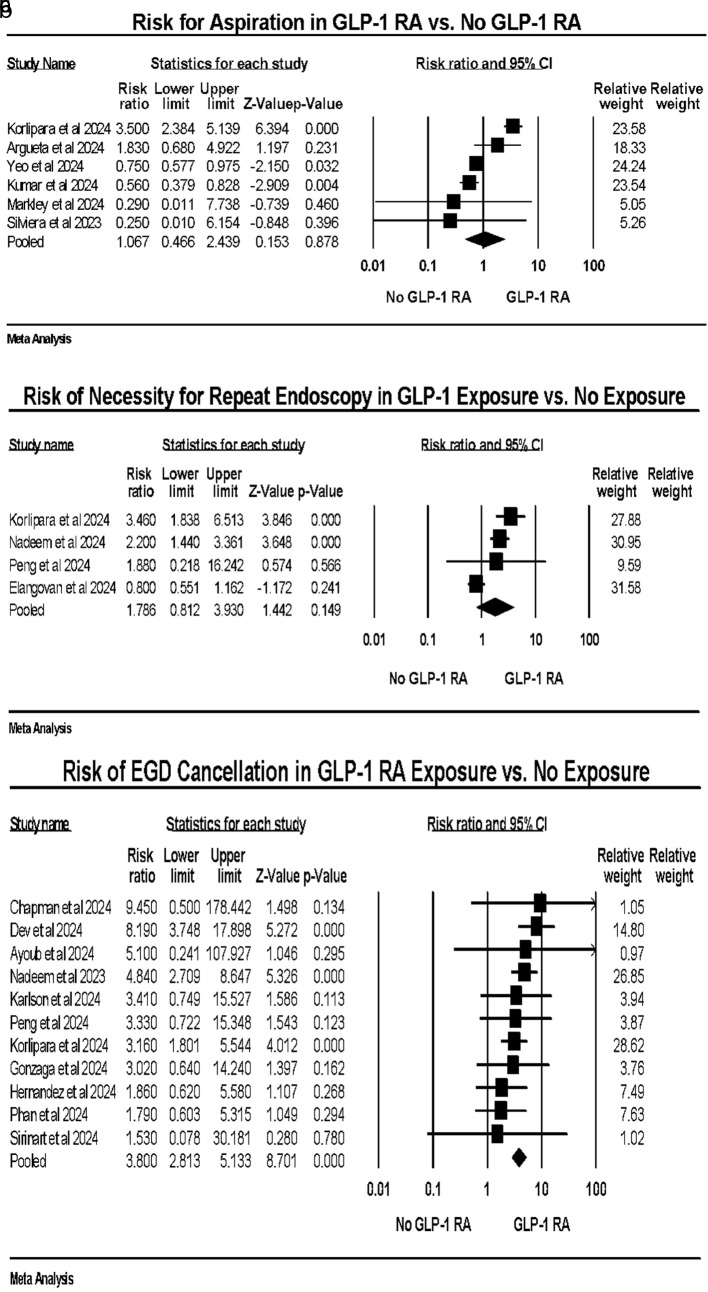
(a) Aspiration events. (b) Repeat upper endoscopy. (c) Cancellation of upper endoscopy.

GLP-1 RA exposure was not associated with aspiration (OR: 1.06, CI: 0.466–2.439, P = 0.145, I^2^: 91.44%) (Fig. 3a) or increased risk of the need for repeat upper endoscopy (OR: 1.78, CI: 0.812–3.930, P = 0.145, I^2^: 85.32%) (Fig. 3b). GLP-1 RA use was associated with higher number of EGD cancellations (OR: 3.80, CI: 2.813–5.133, P < 0.0001, I^2^: 4.19%) (Fig. 3c). Aspiration events were rare across studies, generally occurring in less than 1% of procedures in both groups.

### Quality assessment

Bias assessment for including observational studies using Newcastle-Ottawa scale showed a score of 6–8 for all studies (Supplementary Material 4, gr.elmerpub.com).

### Validation of meta-analysis results

Our sensitivity analysis conducted by excluding abstracts from the RGC outcome revealed a pooled OR of 4.30 (CI: 1.98-9.342), n=, p < 0.0001, I^2^ = 78.7%, n = 8, (Supplementary Material 6a, gr.elmerpub.com). Also, sensitivity analysis performed by excluding one study sequentially did not change the effect size for RGC and cancelled procedures (Supplementary Material 6b, c, gr.elmerpub.com).

### Publication bias assessment

In terms of quantitative publication bias, the Egger’s regression test for RGC and canceled upper endoscopy revealed two-tailed P-value > 0.05 indicating no significant asymmetry for observed studies. Qualitative assessment of funnel plot for RGC and cancellation of upper endoscopy revealed most effect sizes within the expected range of variation. There was some asymmetry with more studies towards the right (Supplementary Material 5, gr.elmerpub.com).

## Discussion

Our primary outcome analysis revealed significantly increased odds of RGC in patients using GLP-1 RAs compared to those not using these agents. The pooled OR of 3.955 (CI: 2.691–5.815) underscores a substantial association, suggesting that GLP-1 RAs significantly contribute to the presence of RGC during endoscopic procedures. This finding is critical, as RGC can impair visibility and therefore limit the procedure, potentially leading to diagnostic inaccuracies and procedural delays. The high heterogeneity (I^2^ = 82.71%) observed in our analysis highlights the variability among the included studies, which may be attributed to differences in study populations, GLP-1 RA types, and study designs.

Our findings are consistent with other studies that have reported an association between GLP-1 RA use and increased gastric content during endoscopy. For instance, Kobori et al [8] found that patients on GLP-1 RAs had a higher incidence of RGC, which impacted the quality of endoscopic examinations. Similarly, Ghazanfar et al [46] observed that GLP-1 RA users were more likely to experience procedural complications due to residual gastric material. These studies reinforce the need for careful management of patients on GLP-1 RAs to mitigate risks associated with endoscopic procedures.

For secondary outcomes, our analysis did not demonstrate a statistically significant increase in aspiration events among patients receiving GLP-1 RAs compared to non-users. However, aspiration events were rare across included studies, and substantial heterogeneity was observed (I^2^ >90%). Therefore, the absence of a statistically significant association should be interpreted cautiously, as this analysis may be underpowered to detect small but clinically meaningful differences in aspiration risk. Further studies are needed to clarify this relationship. This result aligns with studies such as those by Yeo et al [18] and Barlowe et al [47] which also found no significant difference in aspiration events among patients using GLP-1 RAs compared to non-users. These studies indicate that while GLP-1 RAs are associated with other procedural risks, their impact on aspiration events may be minimal or influenced by other confounding factors. Further research is essential to explore these variables and develop a comprehensive understanding of aspiration risk in patients on GLP-1 RAs.

The risk of needing a repeat endoscopy did not meaningfully differ in GLP-1 RA users when compared to non-GLP-1 RA users, with a pooled OR of 1.822 (CI: 0.813–4.085); this did not reach statistical significance (P = 0.145). The increased need for repeat procedures could be related to the presence of RGC, which compromises initial endoscopy quality. This finding emphasizes the importance of optimizing patient preparation protocols to reduce the likelihood of repeat procedures and associated healthcare costs. Consistent with our findings, Stark et al [10] observed an elevated risk of repeat endoscopy in patients on GLP-1 RAs due to poor visualization conditions during the initial procedure. Furthermore, Nadeem et al [37] highlighted the economic implications of repeat endoscopies, emphasizing the need for better pre-procedure management to prevent additional healthcare expenditures. These studies reinforce the necessity for tailored patient preparation protocols for those on GLP-1 RAs.

Notably, the risk of EGD cancellation was significantly higher in patients on GLP-1 RAs, with a pooled OR of 3.966, CI: 2.870–5.563 and minimal observed heterogeneity (I^2^ = 4.14%). This suggests a consistent and strong association across studies, indicating that GLP-1 RA use is a substantial risk factor for EGD cancellation. This outcome is particularly concerning as it directly impacts patient safety and procedural success rates and has downstream effects on financial aspects of endoscopy, patient and physician workflow, and other relevant issues. Consistent with our findings, Mizubuti et al [48] reported a higher rate of EGD cancellations in patients on GLP-1 RAs due to inadequate gastric emptying. Similarly, Peng et al [40] found that the use of GLP-1 RAs significantly increased the likelihood of procedure cancellations, highlighting the need for improved management strategies for these patients.

This is one of the most extensive systematic reviews and meta-analyses on this topic, encompassing 28 retrospective studies with a large sample size of 212,082 patients. This comprehensive approach enhances the generalizability and robustness of our findings. Furthermore, by including both cohort studies and case-control studies, our analysis benefits from a variety of perspectives and methodologies, which adds depth and reliability to our conclusion. Finally, sensitivity analysis that excludes abstracts strengthens the validity of our findings by addressing potential biases and confirming the robustness of the primary outcome. The use of propensity-matched cohorts when available strengthens internal validity but does not eliminate residual confounding.

Our meta-analysis does have a few limitations. First, the reliance on retrospective studies introduces inherent biases, such as recall bias and selection bias, which can affect the accuracy and reliability of the results. Prospective studies are needed to validate these findings. Second, the high heterogeneity observed in some analyses (e.g., RGC and aspiration events) suggests variability in study populations, GLP-1 RA types, dosages, and study designs. This heterogeneity may limit the applicability of the pooled results to specific clinical settings. Although subgroup analyses or meta-regression could further explore sources of heterogeneity, these were not feasible due to inconsistent reporting across studies and lack of stratified data. This limits the ability to identify specific drivers of variability in the pooled estimates.

Third, the studies included in our analysis did not consistently differentiate between different GLP-1 RAs. Variations in the pharmacokinetics and pharmacodynamics of different GLP-1 RAs could influence the outcomes, and further research is needed to explore these differences. Fourth, the potential for publication bias exists, as studies with significant findings are more likely to be published. This bias could skew the results of the meta-analysis and overestimate the risks associated with GLP-1 RA use. Fifth, the quality of the studies included varies, and some studies may have methodological weaknesses that could impact the overall findings. Sixth, the absence of RCTs in our analysis limits the strength of the evidence. Last, several studies included in our analysis are only available as abstracts, and the full manuscripts are not accessible. This limitation may impact the depth of data extraction and the overall quality of evidence, as abstracts often provide limited information compared to full-text articles. Additionally, definitions of retained gastric content were not uniform across studies, which may have contributed to the observed heterogeneity and limit direct comparability of results. Also, timing of the last GLP-1 RA dose prior to endoscopy was inconsistently reported, which may influence gastric emptying and contribute to heterogeneity in outcomes. Additionally, included studies reported a mix of adjusted and unadjusted estimates; pooling these may introduce methodological variability and does not fully account for residual confounding.

From a practical standpoint, these findings have important implications for endoscopists and anesthesia providers. Given the increased likelihood of RGCs and procedure cancellation among patients receiving GLP-1 RAs, pre-procedural assessment should include routine screening for GLP-1 RA use and evaluation for symptoms suggestive of delayed gastric emptying, such as nausea, vomiting, or early satiety. At the time of scheduling, consideration may be given to procedure timing relative to GLP-1 RA dosing, particularly for long-acting agents, and adherence to evolving multi-society guidance regarding temporary withholding of these medications when clinically appropriate.

On the day of the procedure, heightened awareness of potential RGCs is warranted, even in patients who have adhered to standard fasting protocols. In selected patients, particularly those with symptoms or high-risk features, clinicians may consider delaying the procedure, extending fasting duration, or modifying anesthesia approach. However, given the limited prospective evidence, peri-procedural management should remain individualized until more definitive guidance is available.

In summary, our meta-analysis highlights several critical considerations regarding the use of GLP-1 RAs in patients undergoing upper endoscopy. The increased risk of RGC and EGD cancellation associated with GLP-1 RA use underscores the need for careful patient management and potentially modifying pre-endoscopy protocols for these patients.

### Conclusions

#### What is already known on this topic

Given the rising prevalence of GLP-1 RA use and the critical importance of patient safety during upper endoscopic procedures, a systematic review and meta-analysis was needed to rigorously evaluate the potential impact of GLP-1 RAs on endoscopic outcomes. Our study aims to provide a clearer understanding of the risks associated with GLP-1 RA use in the context of upper endoscopy, addressing previous uncertainties and informing clinical decision-making. By synthesizing data from multiple studies, our analysis seeks to guide healthcare providers in balancing the benefits of GLP-1 RAs with potential procedural risks.

#### What this study adds

This study reveals that GLP-1 RAs significantly increase the odds of RGCs and procedure cancellations during upper endoscopy. However, they do not increase the risk of aspiration or the need for repeated procedures. These findings clarify the specific risks associated with GLP-1 RA use before endoscopy, guiding safer clinical practices.

#### How this study might affect research, practice, or policy

This study highlights the need for careful pre-endoscopy assessment of patients on GLP-1 RAs, potentially leading to revised guidelines to minimize procedure cancellations. It encourages further research into alternative management strategies to mitigate risks associated with gastric retention. Policymakers might consider these findings when updating protocols for endoscopic procedures in patients using GLP-1 RAs.

## Supplementary Material

Suppl 1 Full search strategies.

Suppl 2 PRISMA guidelines.

Suppl 3 Baseline characteristics of all studies included in systematic review of GLP-1 RA vs. no GLP-1 RA on upper endoscopy.

Suppl 4 Newcastle-Ottawa scale for quality assessment.

Suppl 5 Publication bias.

Suppl 6 Sensitivity analysis.

## Data Availability

The authors declare that data supporting the findings of this study are available within the article.
