# Supplementary material for: Evaluating Glucagon-Like Peptide-1 Receptor Agonist Safety Before Upper Endoscopy: A Systematic Review and Meta-Analysis
Source: Gastroenterology Res. 2026 Apr 27;19(2):64–73. doi: 10.14740/gr2108 (PMC13171266; doi:10.14740/gr2108)
Supplement: Suppl 1 — Full search strategies. [file gr-19-02-064-s001.docx]

**Suppl 1.** Full search strategies (all searched performed on June 25, 2024).

**Embase (Embase.com, Elsevier)**

| **No.** | **Query** | **Results** |
| --- | --- | --- |
| #1 | 'glucagon like peptide 1 receptor agonist'/syn OR 'tirzepatide'/syn OR 'albiglutide'/syn OR 'beinaglutide'/syn OR 'exendin 4'/syn OR 'liraglutide'/syn OR 'lixisenatide'/syn OR 'semaglutide'/syn OR 'glp 1 agonist*' OR 'glp 1 receptor agonist*' OR 'glucagon like peptide 1 agonist*' OR 'glucagon like peptide 1 receptor stimulating agent*' OR 'glucagon like peptide 1 receptor agonist*' OR 'incretin mimetic*' OR 'long acting glp 1 agonist*' OR 'long acting glp 1 receptor agonist*' OR 'long acting glucagon like peptide 1 agonist*' OR 'long acting glucagon like peptide 1 receptor agonist*' OR tirzepatide OR zepbound OR albiglutide OR beinaglutide OR 'exendin 4' OR byetta OR bydureon OR liraglutide OR victoza OR saxenda OR lixisenatide OR lyxumia OR adlyxin OR semaglutide OR ozempic OR wegovy OR rybelsus | 55461 |
| #2 | 'esophagogastroduodenoscopy'/syn AND 'esophagoscopy'/syn OR 'gastroscopy'/syn OR 'duodenoscopy'/syn OR esophagogastroduodenoscop* OR oesophagogastroduodenoscop* OR egd OR esophagoscop* OR oesophagoscop* OR 'esophagus endoscop*' OR gastroscop* OR 'stomach endoscop*' OR duodenoscop* OR 'duodenum endoscop*' OR 'upper gi endoscop*' OR 'upper endoscop*' OR 'upper gastrointestinal endoscop*' OR 'upper gi tract endoscop*' OR 'upper gastrointestinal tract endoscop*' OR (('upper' AND (gastrointestin* OR 'gastro intestin*' OR 'gastrointestinal endoscopy'/de OR 'digestive tract endoscopy'/de) OR esophag* OR oesophag* OR gastric* OR stomach* OR duoden* OR 'upper gastrointestinal tract'/syn) AND endoscop*) | 269635 |
| #3 | #1 AND #2 | 586 |
| #4 | 'visibility'/syn OR visib* OR polprep | 249490 |
| #5 | 'stomach mucosa'/syn OR mucosa* OR 'mucous membrane*' | 551213 |
| #6 | #4 AND #5 | 5232 |
| #7 | 'stomach content'/syn OR 'stomach emptying'/syn OR 'gastric content*' OR 'stomach content*' OR 'stomach empty*' OR 'stomach evacuat*' OR 'gastric empty*' OR 'gastric evacuat*' OR 'stomach cleanliness*' OR 'stomach retention*' OR 'gastric cleanliness*' OR 'gastric retention*' OR 'content retention*' OR 'retained content*' OR 'retained food*' OR 'food retention*' OR 'food content*' | 40365 |
| #8 | #3 AND #6 | 4 |
| #9 | #3 AND #7 | 171 |
| #10 | #8 OR #9 | 171 |
| #11 | #10 NOT ([animals]/lim NOT [humans]/lim) NOT ('conference review'/it OR 'editorial'/it OR 'letter'/it OR 'note'/it OR 'review'/it OR 'short survey'/it OR 'tombstone'/it OR 'case report'/de OR 'meta analysis'/de OR 'meta analysis topic'/de OR 'systematic review'/de OR 'systematic review topic'/de) | 107 |

**MEDLINE (PubMed, National Center for Biotechnology Information, National Library of Medicine)**

| **No.** | **Query** | **Results** |
| --- | --- | --- |
| 1 | "Glucagon-Like Peptide-1 Receptor Agonists"[Mesh] OR "Liraglutide"[Mesh] OR "tirzepatide"[Supplementary Concept] OR "Exenatide"[Mesh] OR "lixisenatide"[Supplementary Concept] OR GLP-1-agonist* OR GLP-1-receptor-agonist* OR glucagon-like-peptide-1-agonist* OR glucagon-like-peptide-1-receptor-stimulating-agent* OR glucagon-like-peptide-1-receptor-agonist* OR incretin-mimetic* OR long-acting-GLP-1-agonist* OR long-acting-GLP-1-receptor-agonist* OR long-acting-glucagon-like-peptide-1-agonist* OR long-acting-glucagon-like-peptide-1-receptor-agonist* OR tirzepatide OR zepbound OR albiglutide OR beinaglutide OR exendin-4 OR Byetta OR Bydureon OR liraglutide OR Victoza OR Saxenda OR lixisenatide OR Lyxumia OR Adlyxin OR semaglutide OR Ozempic OR Wegovy OR rybelsus | 15,432 |
| 2 | "Duodenoscopy"[Mesh] OR "Gastroscopy"[Mesh] OR "Esophagoscopy"[Mesh] OR Esophagogastroduodenoscop* OR oesophagogastroduodenoscop* OR EGD OR esophagoscop* OR oesophagoscop* OR esophagus-endoscop* OR gastroscop* OR stomach-endoscop* OR duodenoscop* OR duodenum-endoscop* OR upper-GI-endoscop* OR upper-endoscop* OR upper-gastrointestinal-endoscop* OR upper-gi-tract-endoscop* OR upper-gastrointestinal-tract-endoscop* OR ((("Upper" AND (gastrointestin* OR gastro-intestin* OR "Endoscopy, Digestive System"[Mesh:NoExp] OR "Endoscopy, Gastrointestinal"[Mesh:NoExp]) ) OR esophag* OR oesophag* OR gastric* OR stomach* OR duoden*) AND endoscop*) | 129,088 |
| 3 | #1 AND #2 | 55 |
| 4 | "Gastric Mucosa"[Mesh] OR Mucosa* OR mucous-membrane* | 415,053 |
| 5 | Visib* OR POLPREP | 196,739 |
| 6 | #4 AND #5 | 2,868 |
| 7 | "Gastrointestinal Contents"[Mesh] OR "Gastric Emptying"[Mesh] OR Gastric-content* OR stomach-content* OR stomach-empty* OR stomach-evacuat* OR gastric-empty* OR gastric-evacuat* OR stomach-cleanliness* OR stomach-retention* OR gastric-cleanliness* OR gastric-retention* OR content-retention* OR retained-content* OR retained-food* OR food-retention* OR food-content* | 27,183 |
| 8 | #3 AND #6 | 1 |
| 9 | #3 AND #7 | 25 |
| 10 | #8 OR #9 | 25 |
| 11 | #10 NOT ("animals"[mesh] NOT "humans"[mesh]) NOT ("case reports"[Publication Type] OR "comment"[Publication Type] OR "editorial"[Publication Type] OR "guideline"[Publication Type] OR "introductory journal article"[Publication Type] OR "meta analysis"[Publication Type] OR "news"[Publication Type] OR "retracted publication"[Publication Type] OR "review"[Publication Type] OR "systematic review"[Publication Type]) | 16 |

**Cochrane Central Register of Controlled Trials (CochraneLibrary.com platform, Wiley, Issue 5 of 12, May 2024)**

| **ID** | **Search** | **Hits** |
| --- | --- | --- |
| #1 | [mh "Glucagon-Like Peptide-1 Receptor Agonists"] OR [mh "Liraglutide"] OR [mh "Exenatide"] OR "GLP 1 agonists" OR "GLP 1 receptor agonists" OR "glucagon like peptide 1 agonists" OR "glucagon like peptide 1 receptor stimulating agent" OR "glucagon like peptide 1 receptor agonists" OR "GLP 1 agonist" OR "GLP 1 receptor agonist" OR "glucagon like peptide 1 agonist" OR "glucagon like peptide 1 receptor stimulating agent" OR "glucagon like peptide 1 receptor agonist" OR incretin mimetic* OR tirzepatide OR zepbound OR albiglutide OR beinaglutide OR "exendin 4" OR Byetta OR Bydureon OR liraglutide OR Victoza OR Saxenda OR lixisenatide  OR Lyxumia OR Adlyxin OR semaglutide OR Ozempic OR Wegovy OR rybelsus | 5689 |
| #2 | [mh "Duodenoscopy"] OR [mh "Gastroscopy"] OR [mh "Esophagoscopy"] OR Esophagogastroduodenoscop* OR oesophagogastroduodenoscop* OR EGD OR esophagoscop* OR oesophagoscop* OR esophagus-endoscop* OR gastroscop* OR stomach-endoscop* OR duodenoscop* OR duodenum-endoscop* OR upper-GI-endoscop* OR upper-endoscop* OR upper-gastrointestinal-endoscop* OR upper-gi-tract-endoscop* OR upper-gastrointestinal-tract-endoscop* OR ((("Upper" AND (gastrointestin* OR gastro-intestin* OR [mh ^"Endoscopy, Digestive System"] OR [mh ^"Endoscopy, Gastrointestinal"]) ) OR esophag* OR oesophag* OR gastric* OR stomach* OR duoden*) AND endoscop*) | 16056 |
| #3 | #1 AND #2 | 20 |
| #4 | [mh "Gastric Mucosa"] OR Mucosa* OR mucous-membrane* | 24859 |
| #5 | Visib* OR POLPREP | 8637 |
| #6 | #4 AND #5 | 643 |
| #7 | [mh "Gastrointestinal Contents"] OR [mh "Gastric Emptying"] OR Gastric-content* OR stomach-content* OR stomach-empty* OR stomach-evacuat* OR gastric-empty* OR gastric-evacuat* OR stomach-cleanliness* OR stomach-retention* OR gastric-cleanliness* OR gastric-retention* OR content-retention* OR retained-content* OR retained-food* OR food-retention* OR food-content* | 5631 |
| #8 | #3 AND #6 | 0 |
| #9 | #3 AND #7 | 6 |
| #10 | #8 OR #9 | 6 |
| #11 | #10 in Trials | 6 |

**Web of Science Core Collection (Web of Science Platform, Clarivate, Editions = Arts & Humanities Citation Index, Emerging Sources Citation Index, Conference Proceedings Citation Index, Science Citation Index-EXPANDED, and Social Science Citation Index)**

| GLP-1-agonist* OR GLP-1-receptor-agonist* OR glucagon-like-peptide-1-agonist* OR glucagon-like-peptide-1-receptor-stimulating-agent* OR glucagon-like-peptide-1-receptor-agonist* OR incretin-mimetic* OR long-acting-GLP-1-agonist* OR long-acting-GLP-1-receptor-agonist* OR long-acting-glucagon-like-peptide-1-agonist* OR long-acting-glucagon-like-peptide-1-receptor-agonist* OR tirzepatide OR zepbound OR albiglutide OR beinaglutide OR exendin-4 OR Byetta OR Bydureon OR liraglutide OR Victoza OR Saxenda OR lixisenatide OR Lyxumia OR Adlyxin OR semaglutide OR Ozempic OR Wegovy OR rybelsus (Topic)  and  Esophagogastroduodenoscop* OR oesophagogastroduodenoscop* OR EGD OR esophagoscop* OR oesophagoscop* OR esophagus-endoscop* OR gastroscop* OR stomach-endoscop* OR duodenoscop* OR duodenum-endoscop* OR upper-GI-endoscop* OR upper-endoscop* OR upper-gastrointestinal-endoscop* OR upper-gi-tract-endoscop* OR upper-gastrointestinal-tract-endoscop* OR ((("Upper" AND (gastrointestin* OR gastro-intestin*) ) OR esophag* OR oesophag* OR gastric* OR stomach* OR duoden*) AND endoscop*) (Topic)  and  (Gastric-content* OR stomach-content* OR stomach-empty* OR stomach-evacuat* OR gastric-empty* OR gastric-evacuat* OR stomach-cleanliness* OR stomach-retention* OR gastric-cleanliness* OR gastric-retention* OR content-retention* OR retained-content* OR retained-food* OR food-retention* OR food-content*) OR ((Visib* OR POLPREP) AND (Mucosa* OR mucous-membrane*)) (Topic)  and  Preprint Citation Index (Exclude – Database) and Case Report or Editorial Material or Review Article or Awarded Grant or Letter (Exclude – Document Types) and Web of Science Core Collection (Database) | 14 |
| --- | --- |
