# Supplementary material for: Evaluating Glucagon-Like Peptide-1 Receptor Agonist Safety Before Upper Endoscopy: A Systematic Review and Meta-Analysis
Source: Gastroenterology Res. 2026 Apr 27;19(2):64–73. doi: 10.14740/gr2108 (PMC13171266; doi:10.14740/gr2108)
Supplement: Suppl 3 — Baseline characteristics of all studies included in systematic review of GLP-1 RA vs. no GLP-1 RA on upper endoscopy. [file gr-19-02-064-s003.docx]

**Suppl 3.** Baseline characteristics of all studies included in systematic review of GLP-1 RA vs. no GLP-1 RA on upper endoscopy.

| **Study, year** | **Study design** | **M/A** | **Years** | **Raw total sample size** | **Analyzed sample size** | **n** | | **Source** | **City, State, Country** | **Propensity matched** | **GLP-1 RA** | **Procedure** | **Age** | **% Female** | **BMI ± SD or range (average for whole study)** | **BMI/weight ± SD or range** | | **% Population with DM** | **% Population with DM between groups** | |
| --- | --- | --- | --- | --- | --- | --- | --- | --- | --- | --- | --- | --- | --- | --- | --- | --- | --- | --- | --- | --- |
|  |  |  |  |  |  | **GLP-1** | **No** |  |  |  |  |  |  |  |  | **GLP-1** | **No** |  | **GLP-1** | **No** |
| Argueta et al 2024 [24] | CH | A | 2020-2022 | 41,367 | 1,042 | 571 | 571 | TriNetX | NR | Yes | NR | EGD | 59 | 56.3 |  | - | - |  | - | - |
| Ayoub et al 2024 [25] | CH | A | NR | 205 | 205 | 101 | 104 | SCRCR | Charleston, WV | No | NR | Pre-bariatric EGD | 39 | 81.0 | 38.70 ± 10.81 | No significant difference | |  | - | - |
| Chapman et al 2024 [17] | CC | M | 2017-2023 | 7,735 | 168 | 84 | 84 | SCRCR | Birmingham, AL | Yes | Mix | EGD | 54 | 70.2 | 35.96 ± 12.91 | 40.7 ± 13.29 | 31.23 ± 10.65 | 85.70% | Baseline similar | |
| Dev et al 2024 [27] | CH | A | 2018-2023 | 32,225 | 32,225 | 1,023 | 31,202 | MCRCR | New York City, NY | No | Mix | EGD 82.7%, ERCP 4.3%, upper EUS 12.2%, push enteroscopy 0.8%. | 60 | 50.9 | 33.2 ± 8.2 | No significant difference | | 84.40% | Baseline NR | |
| Elangovan et al 2024 [28] | CH | A | 2014-2023 | 262,290 | 4,398 | 2,199 | 2,199 | TriNetX | Cambridge, MA | Yes | NR | EGD | 47 | 87.4 | NR | No significant difference | | 0% | Baseline similar | |
| Essop et al 2024 [26] | CH | A | 2019-2023 | 576 | 576 | 45 | 531 | SCRCR | Danville, PA | No | NR | EGD | 43 | 83.0 | 46.2 | Not reported | | 27% | Baseline NR | |
| Garza et al 2024 [29] | CC | A | 2018-2023 | 16,295 | 612 | 306 | 306 | SCRCR | St. Louis, MO | Yes | NR | EGD | 61 | 50% | 32.6 (28.2–38) | No significant difference | | 89% | Baseline similar | |
| Gonzaga et al 2024 [30] | CH | A | 2023 | 1,438 | 1,046 | 73 | 973 | SCRCR | Florida | No | NR | EGD | 56 | 64.50% | 28 | 34.4 (28.6–39) | 27.7 (24.4–32.2) | 17.60% | 72.60% | 13.50% |
| Gu et al 2024 [31] | CC | A | 2022-2023 | NR | 304 | 152 | 152 | SCRCR | Boston, MA | Yes | SG | EGD | 58 | - | 35 | 34.4 ± 6.9 | 35.5 ± 6.6 | NR | - | - |
| Hernandez et al 2024 [32] | CH | A | 2022-2023 | 1,167 | 1,058 | 109 | 949 | SCRCR | Chicago, IL | No | NR | EGD | - | - |  | - | - |  | - | - |
| Karlson et al 2024 [33] | CC | A | 2015-2023 | 860 | 860 | 579 | 281 | SCRCR | Boston, MA | No | NR | EGD | 60 | 63.0 |  | - | - | 72 | - | - |
| Kobori et al 2023 [8] | CC | M | 2020-2022 | 1,128 | 410 | 205 | 205 | SCRCR | Tokyo, Japan | Yes | NR | EGD | 71 | 22.70% |  | - | - | 100% | - | - |
| Korlipara et al 2024 [34] | CH | A | 2018-2023 | 1,212 | 1,212 | 603 | 609 | SCRCR | New York City, NY | No | SG | EGD | 59 | 69.70% |  | - | - | 45.80% | 54.50% | 37.10% |
| Kumar et al 2024 [43] | CH | A | NR | 122,646 | 122,646 | 61,355 | 61,291 | TriNetX | NR | Yes | Mix | EGD | 62 | 56.50% |  | - | - |  | - | - |
| Markley et al 2024 [35] | CH | A | 2020-2023 | 371 | 371 | 47 | 324 | SCRCR | New York City, NY | No | NR | EGD | 43 | 60.10% | 27.71 | 32.88 ± 6.25 | 26.94 ± 6.37 | 13.80% | 48.90% | 8.60% |
| Meluban et al 2024 [36] | CH | A | 2022-2023 | 2,110 | 2,110 | 65 | 2,045 | SCRCR | Cleveland, OH | No | Mix | EGD | - | 58.50% | NR | - | - |  | - | - |
| Nadeem et al 2023 [37] | CH | M | 2019-2023 | 34,261 | 6,163 | 756 | 5,407 | SCRCR | Danville, PA | Yes | NR | EGD | - | - |  | - | - |  | Baseline similar | |
| Nasser et al 2024 [38] | CC | A | 2023 | 209 | 72 | 48 | 24 | SCRCR | NR | No | NR | EGD | 63 | 51.6%% | 33.59 ± 6.36. | 34.4 ± 7.2 | 33.2 ± 5.9 | 47.85% | 82.90% | 30.20% |
| Panchal et al 2024 [39] | CH | A | 2022-2023 | 599 | 598 | 360 | 238 | SCRCR | Philadelphia, PA | No | NR | EGD | 60 | 61% |  | - | - |  | 68% | 57% |
| Peng et al 2024 [40] | CC | A | 2023 | 153 | 153 | 105 | 49 | SCRCR | Houston, TX | No | NR | EGD | 57 | 73.20% | 35.71 ± 8.92 | 37 ± 10 | 33 ± 6 | 86.27% | 89 | 80 |
| Phan et al 2025 [41] | CS | M | 2021-2023 | 815 | 815 | 409 | 416 | MCRCR | NR | No | Mix | EGD | 61 | 58% |  | - | - | 81% | 93% | 80% |
| Rizvi et al 2024 [42] | CH | A | 2021-2023 | 2,646 | 2,646 | 217 | 2,429 | MCRCR | NR | Yes | SG | EGD | 55 | 59.60% |  | - | - |  | - | - |
| Silviera et al 2023 [9] | CH | M | 2021-2022 | 404 | 404 | 33 | 371 | SCRCR | Sao Paolo, Brazil | Yes | SG | EGD | 50 | 48.50% | 26.2 (22.98–28.73) | 27.0 (25.1–32.8) | 26.1 (23–28.7) | 9.40% | - | - |
| Siranart et al 2024 [44] | CH | A | 2022-2023 | 143 | 143 | 33 | 110 | SCRCR | Boston, MA | No | Mix | Pre-bariatric EGD | 49 | 94.00% |  | - | - |  | - | - |
| Stark et al 2022 [10] | CH | M | 2015-2020 | 357 | 177 | 59 | 118 | SCRCR | Fayetteville, AR | Yes | Mix | EGD | 65 | 10% | 33 ± 6 | 33 ± 6 | 33 ± 6 | 97.70% | 97 | 98 |
| Wu et al 2024 [19] | CH | M | 2019-2023 | 220 | 192 | 90 | 102 | SCRCR | Boston, MA | No | NR | EGD | 61 | 57.30% | 34 | No significant difference | | 43.30% | 69% | 25% |
| Yeo et al 2024 [18] | CH | M | 2018-2020 | 30,177 | 30,177 | 3,372 | 3,331 | TriNetX | NR | Yes | NR | EGD | NR | 54.70% | - | - | - | - | - | - |
| Zaffar et al 2024 [45] | CH | A | 2022-2023 | 2,578 | 1,299 | 1,189 | 110 | SCRCR | Baltimore, MD | No | Mix | EGD | NR | - | - | - | - | - | - | - |

TriNetX is a network of healthcare organizations that provide real-world data and evidence for drug development and medical research. NR stands for “not reported” as these studies included all GLP-1 RAs and did not specify any specific drug. A: abstract; BMI: body mass index; CC: case-control; CH: cohort; CS: cross-sectional; EGD: esophagogastroduodenoscopy; ERCP: endoscopic retrograde cholangiopancreatography; EUS: endoscopic ultrasound; GLP-1 RA: glucagon-like peptide-1 receptor agonist; M: manuscript; MCRCR: multi-center retrospective chart review; SCRCR: single-center retrospective chart review; SD: standard deviation; SG: semaglutide.
