# Supplementary material for: Evaluating Glucagon-Like Peptide-1 Receptor Agonist Safety Before Upper Endoscopy: A Systematic Review and Meta-Analysis
Source: Gastroenterology Res. 2026 Apr 27;19(2):64–73. doi: 10.14740/gr2108 (PMC13171266; doi:10.14740/gr2108)
Supplement: Suppl 4 — Newcastle-Ottawa scale for quality assessment. [file gr-19-02-064-s004.docx]

**Suppl 4.** Newcastle-Ottawa scale for quality assessment

| **Study** |  | **Selection** | | | | **Comparability** | **Outcomes** | | | **Total** |
| --- | --- | --- | --- | --- | --- | --- | --- | --- | --- | --- |
|  |  | **Representativeness of exposed cohort** | **Selection of Non-exposed cohort** | **Ascertainment of Exposure** | **Outcome not present at the Start of study** |  | **Assessment of outcomes** | **Length of follow-up** | **Adequacy of follow-up** |  |
| Argueta et al, 2024 [24] |  | * | * | * | * | * | * | * | 0 | 7 |
| Ayoub et al, 2024 [25] |  | * | * | * | * | * | * | * | 0 | 7 |
| Chapman et al, 2024 [17] |  | * | * | * | * | * | 0 | * | * | 7 |
| Dev et al, 2024 [27] |  | * | * | * | * | * | 0 | * | 0 | 6 |
| Elangova et al, 2024 [28] |  | * | * | * | * | * | * | * | * | 8 |
| Essop et al, 2024 [26] |  | * | * | * | * | * | * | * | * | 8 |
| Garza et al, 2024 [29] |  | * | * | * | * | * | * | * | * | 8 |
| Gonzaga et al, 2024 [30] |  | * | * | * | * | * | * | * | * | 8 |
| Gu et al, 2024 [31] |  | * | * | * | * | * | * | * | 0 | 7 |
| Hernandez et al, 2024 [32] |  | * | * | * | * | * | * | * | 0 | 7 |
| Karlson et al, 2024 [33] |  | * | * | * | * | * | * | * | * | 8 |
| Kobori et al, 2023 [8] |  | * | * | * | * | * | * | * | * | 8 |
| Korlipara et al, 2023 [35] |  | * | * | * | * | * | * | * | * | 8 |
| Kumar et al, 2024 [44] |  | * | * | * | * | * | * | * | * | 8 |
| Markley et al, 2024 [36] |  | * | * | * | * | * | * | * | * | 8 |
| Meluban et al, 2024 [37] |  | * | * | * | * | * | 0 | * | * | 7 |
| Nadeem et al, 2023 [38] |  | * | * | * | * | * | 0 | * | * | 7 |
| Nasser et al, 2024 [39] |  | * | * | * | * | * | 0 | * | * | 7 |
| Panchal et al, 2024 [40] |  | * | * | * | * | * | 0 | * | 0 | 6 |
| Peng et al, 2024 [41] |  | * | * | * | * | * | 0 | * | 0 | 6 |
| Phan et al, 2025 [42] |  | * | * | * | * | * | 0 | * | * | 7 |
| Rizvi et al, 2024 [43] |  | * | * | * | * | * | 0 | * | * | 7 |
| Silviera et al, 2023 [44] |  | * | * | * | * | * | * | * | 0 | 7 |
| Siranart et al, 2024 [45] |  | * | * | * | * | * | * | * | 0 | 7 |
| Stark et al, 2022 [10] |  | * | * | * | * | * | * | * | * | 8 |
| Wu et al, 2024 [19] |  | * | * | * | * | * | * |  | * | 8 |
| Yeo et al, 2024 [18] |  | * | * | * | * | * | * |  | * | 8 |
| Zaffar et al, 2024 [46] |  | * | * | * | * | * | * |  | 0 | 7 |
