# Supplementary figures and images for: Evaluating Glucagon-Like Peptide-1 Receptor Agonist Safety Before Upper Endoscopy: A Systematic Review and Meta-Analysis
Source: Gastroenterology Res. 2026 Apr 27;19(2):64–73. doi: 10.14740/gr2108 (PMC13171266; doi:10.14740/gr2108)

**Suppl 5.** Publication bias

1. **RGC funnel plot**


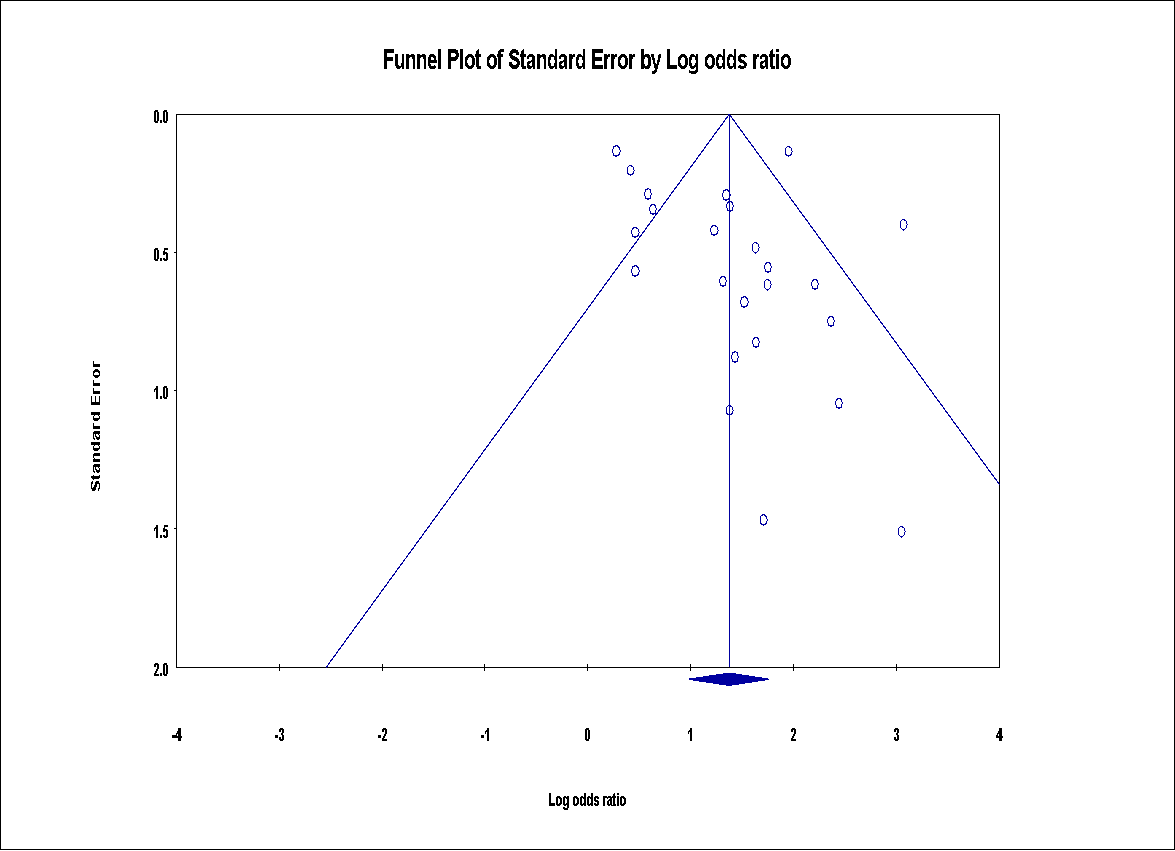


1. **Cancelled procedures funnel plot**


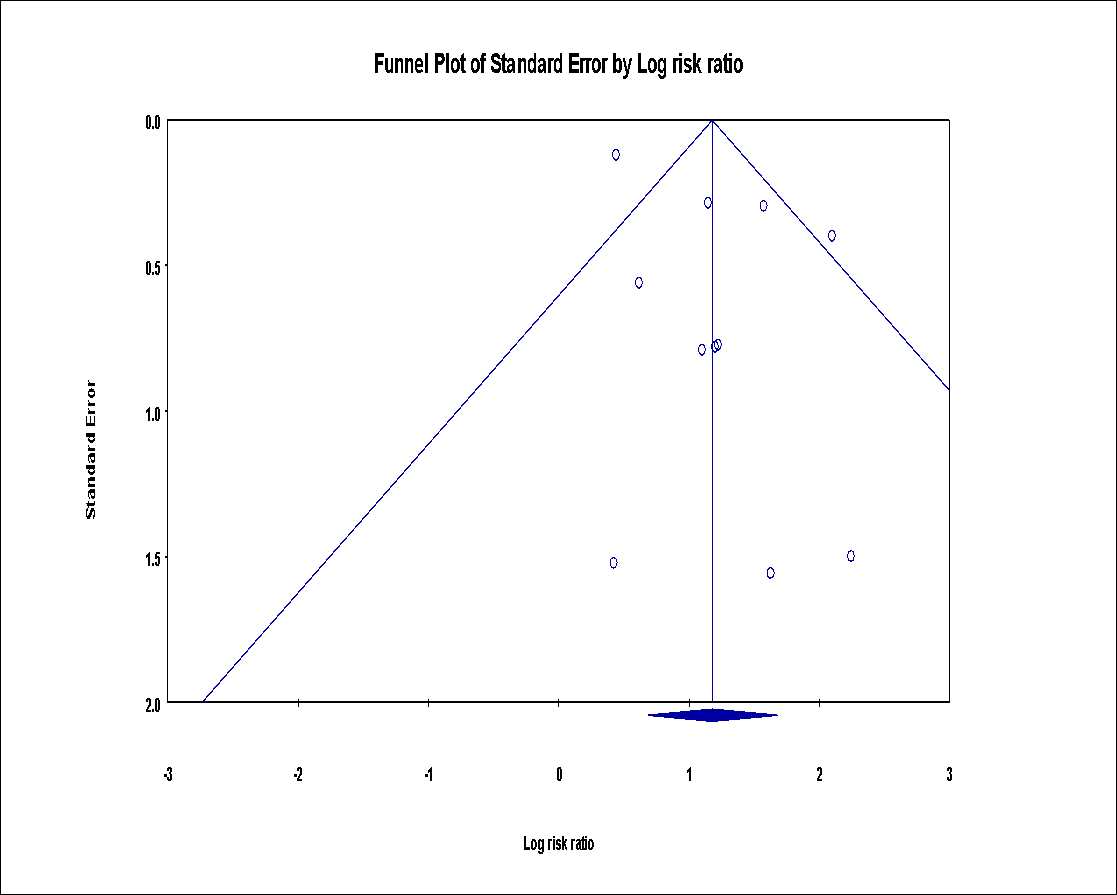

Supplement: Suppl 5 — Publication bias. [file gr-19-02-064-s005.docx]
