# Supplementary material for: Evaluating Glucagon-Like Peptide-1 Receptor Agonist Safety Before Upper Endoscopy: A Systematic Review and Meta-Analysis
Source: Gastroenterology Res. 2026 Apr 27;19(2):64–73. doi: 10.14740/gr2108 (PMC13171266; doi:10.14740/gr2108)

**Suppl 6.** Sensitivity analysis

(a) Sensitivity analysis **excluding abstracts** for RGC


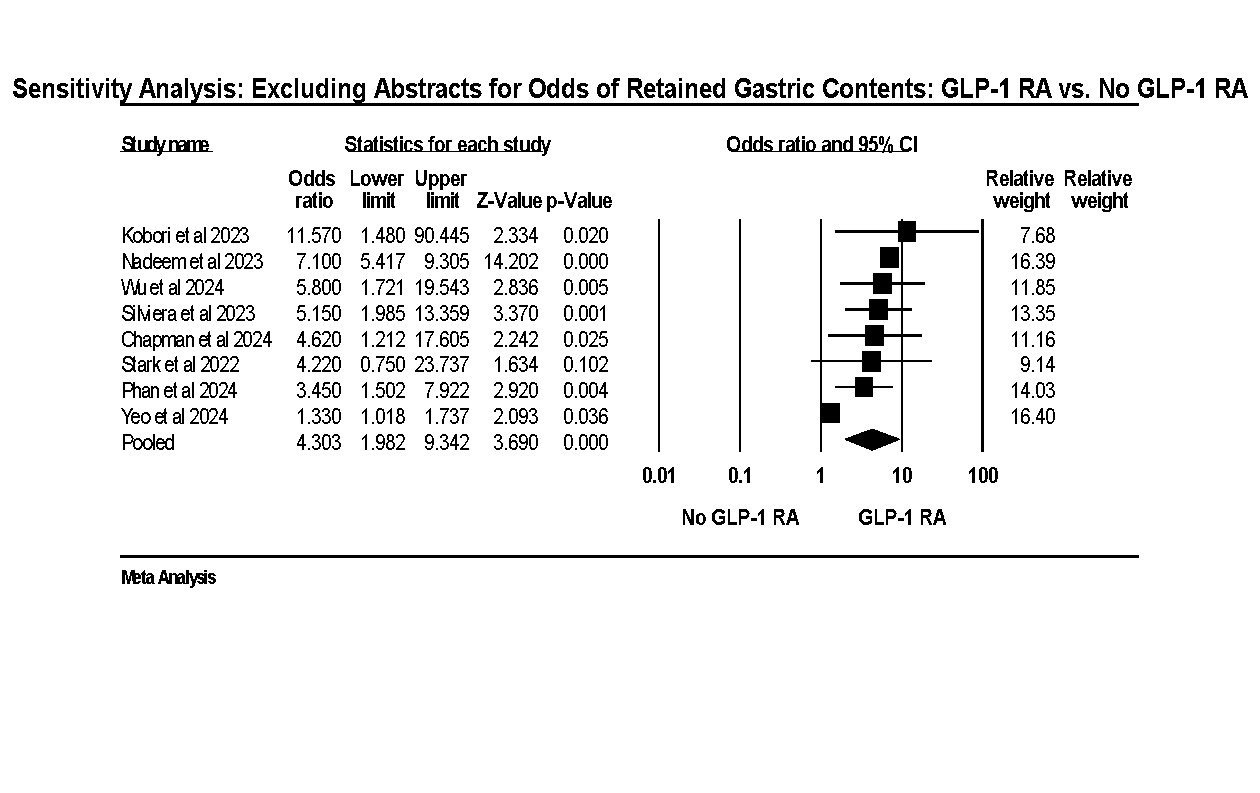


(b) Sensitivity analysis excluding one study for RGC


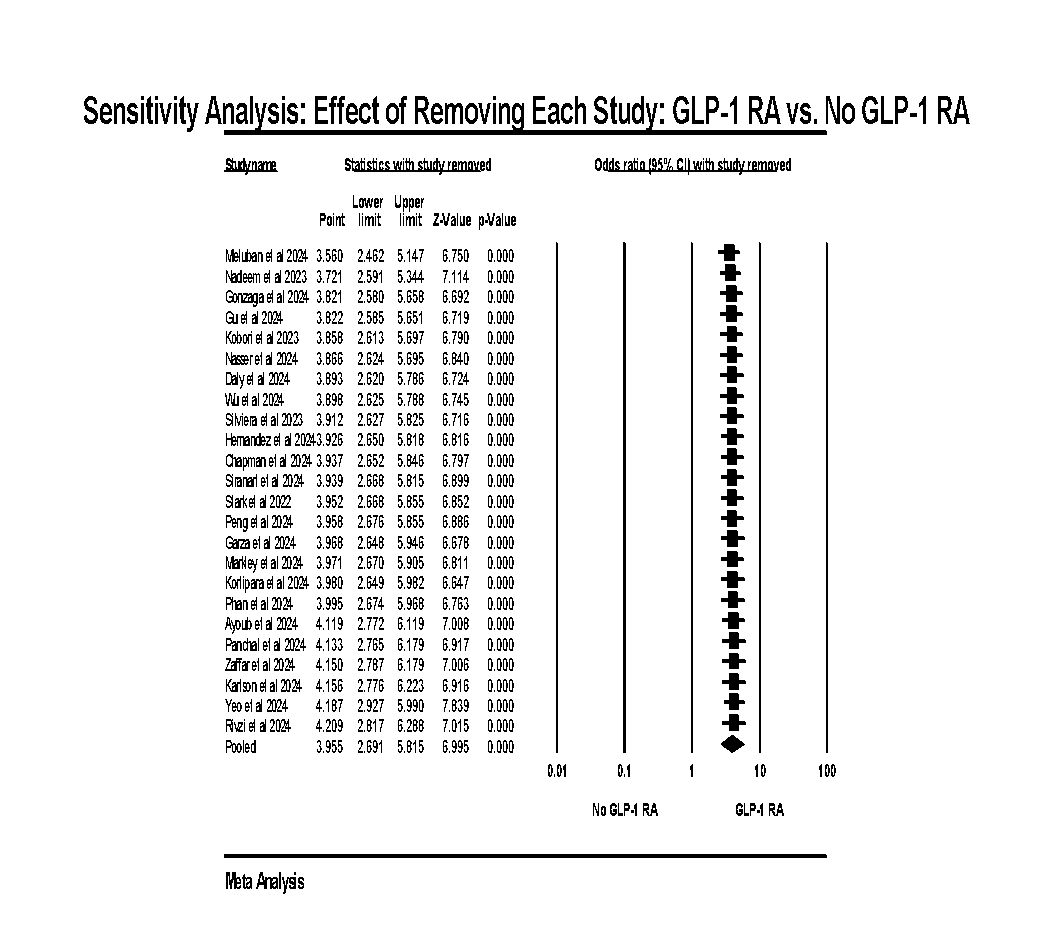


(c) Sensitivity analysis excluding one study for cancelled procedure


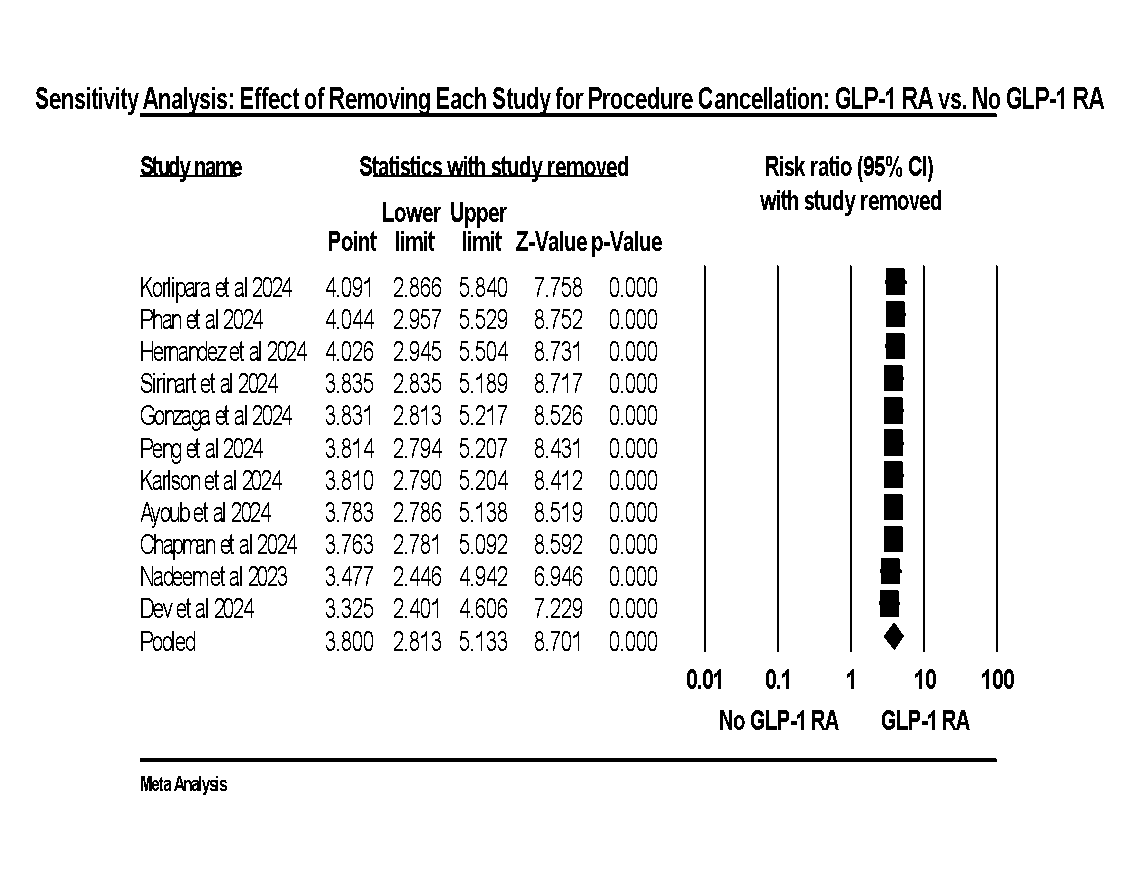

Supplement: Suppl 6 — Sensitivity analysis. [file gr-19-02-064-s006.docx]
